# FACTORS ASSOCIATED WITH INADEQUATE MILK CONSUMPTION AMONG ADOLESCENTS: NATIONAL SCHOOL HEALTH SURVEY - PENSE 2012

**DOI:** 10.1590/1984-0462/2020/38/2018184

**Published:** 2019-11-25

**Authors:** Janiquelli Barbosa Silva, Bianca Caroline Elias, Laís Amaral Mais, Sarah Warkentin, Tulio Konstantyner, Fernanda Luisa Ceragioli Oliveira

**Affiliations:** a Universidade Federal de São Paulo, São Paulo, SP, Brazil.

**Keywords:** Adolescent, Food consumption, Milk, Calcium, Epidemiological surveys, Logistic models, Adolescente, Consumo alimentar, Leite, Cálcio, Inquéritos epidemiológicos, Modelos logísticos

## Abstract

**Objective:**

To identify the prevalence and factors associated with inadequate milk consumption among adolescents.

**Methods:**

This was a cross-sectional study based on secondary data from the National School Health Survey (2012), a Brazilian survey carried out using a self-administered questionnaire in a representative sample of 9^th^-grade students from public and private schools. The frequency of milk intake and its association with socio-demographic characteristics, food consumption and physical activity were estimated. A descriptive and inferential analysis of factors associated with inadequate milk consumption (no consumption at least one of the seven days of the week) was performed. A multiple logistic model was adjusted to control confounders.

**Results:**

The sample included 108,828 adolescents and inadequate milk consumption ocurred in 58.9%. The final model included nine variables independently associated with inadequate milk intake: breakfast frequency less than 4 days per week (odds ratio [OR]=2.40; p<0.001), unprocessed or minimally processed foods intake less than 5 days per week (OR=1.93; p<0.001), living in the northeast region (OR=1.39; p<0.001), less maternal schooling (OR=1.35; p<0.001), physical inactivity (OR=1.33; p<0.001), attending public school (OR=1.26; p<0.001), not being white (OR=1.14; p<0.001), being older than 14 years old (OR=1.13; p<0.001) and having a habit of eating meals while watching TV or studying (OR=1.04; p=0.036).

**Conclusions:**

Inadequate milk consumption is prevalent among Brazilian adolescents. The identification of associated factors suggests the need to develop nutritional guidance strategies for the prevention of diseases that result from low calcium intake.

## RESUMO

**Objetivo:**

Identificar a prevalência e os fatores associados ao consumo inadequado de leite em adolescentes.

**Métodos:**

Estudo transversal com base em dados secundários da Pesquisa Nacional de Saúde do Escolar (PeNSE; 2012), inquérito brasileiro realizado por meio de questionário autoaplicável em amostra representativa de alunos do nono ano do ensino fundamental de escolas públicas e privadas. Estimou-se a frequência da ingestão de leite e sua associação com características sociodemográficas, consumo alimentar e prática de atividade física. Foi realizada análise descritiva e inferencial dos fatores associados ao consumo inadequado de leite (ausência em pelo menos um dos sete dias da semana). Um modelo logístico múltiplo foi ajustado para controle das variáveis de confusão.

**Resultados:**

A amostra incluiu 108.828 adolescentes e o consumo inadequado de leite foi de 58,9%. O modelo final incluiu 9 variáveis independentemente associadas à ingestão inadequada de leite: frequência de desjejum inferior a 4 dias semanais (*odds ratio* [OR]=2,40; p<0,001), consumo de alimentos *in natura* e minimamente processados inferior a 5 dias semanais (OR=1,93; p<0,001), residir na Região Nordeste (OR=1,39; p<0,001), menor escolaridade materna (OR=1,35; p<0,001), inatividade física (OR=1,33; p<0,001), frequentar escola pública (OR=1,26; p<0,001), não ser da raça branca (OR=1,14; p<0,001), ter idade superior a 14 anos (OR=1,13; p<0,001) e possuir o hábito de realizar as refeições assistindo à TV ou estudando (OR=1,04; p=0,036).

**Conclusões:**

O consumo inadequado de leite é frequente entre adolescentes brasileiros. A identificação de fatores associados sugere a necessidade do desenvolvimento de estratégias de orientação nutricional para a prevenção de doenças resultantes da baixa ingestão de cálcio.

## INTRODUCTION

Milk and its derivatives are a food group that has high nutritional value, and it is recommended as part of a balanced diet. Drinking milk is recommended during all stages of life, as it provides indispensable nutrients such as amino acids, fatty acids, minerals (calcium, magnesium, selenium) and vitamins (retinol, cyanocobalamin, pantothenic acid).^1^ ^,^ ^2^ ^,^ ^3^

Calcium present in milk is highly bioavailable, reaching about 70% use value when compared to other foods. This makes it an important part of human nutrition.^2^ ^,^ ^3^ ^,^ ^4^ The micronutrient calcium is one of the most abundant minerals in the human body, found mainly in the structure of bones. Not only does it participate in bone metabolism, but it also has a role in enzymatic and metabolic reactions, in the coagulation process, in the signaling and adhesion of cells, in mediating muscle contractions, in the secretion of hormones and neurotransmitters, and in the transport of substances.^2^ ^,^ ^3^ ^,^ ^5^ Milk is among the most acquired kind of food among Brazilian families. There will be an estimated 23% increase in the production of milk around the world in the next decade, with 73% of this additional supply coming from developing countries.^1^ ^,^ ^2^ ^,^ ^6^

Adolescence, which includes the age group from 10 to 19 years old, is a biopsychosocial maturation cycle characterized by the broad biological development of organs, tissues and systems; and by intense physical, sexual, cognitive, social and emotional changes. This process is directly influenced by the interaction of genetic, environmental and endocrine factors, whose organic and nutritional

demands are increased by accelerated physical growth patterns.^5^ Exclusion or inadequate consumption of milk at this stage impairs structural growth, leads to lower bone mineral density and, consequently, contributes to the development of osteoporosis and increased risk of fractures in adulthood and old age.^2^ ^,^ ^3^ ^,^ ^5^ ^,^ ^7^ Based on data from the National Health and Nutrition Examination Survey, an association was found between lower milk intake during adolescence, lower bone mineral content and lower bone mass among women aged 20 and 49 years old.^7^

In Brazil, studies indicate that there is a significant reduction in milk consumption among adolescents, with an inverse increase in the intake of processed and high sugar drinks. Similarly, such products are among the most consumed beverages by young Europeans, as data from the European Youth Heart Study (EYHS) demonstrate. Such changes in consumption have led to calcium intake below the recommended amount and higher consumption of low nutritional value foods.^8^ ^,^ ^9^ ^,^ ^10^ ^,^ ^11^ ^,^ ^12^ In this context, the objective of the present study was to identify factors associated with inadequate milk consumption among adolescents. Additionally, it aimed to contribute to the development of strategies for control and prevention of nutritional disorders and, consequently, changes in growth and of bone metabolism in this age group.

## METHOD

This study is cross-sectional and used secondary data from the second version of the National School Health Survey (*Pesquisa Nacional de Saúde do Escolar* - PeNSE), conducted between April and September 2012. It is a population-based Brazilian school health survey conducted by the Ministry of Health (*Ministério da Saúde* - MS) in partnership with the Brazilian Institute of Geography and Statistics (*Instituto Brasileiro de Geografia e Estatística* - IBGE). The database is in the public domain and is available electronically on the IBGE website.^13^

The target population consisted of students attending ninth grade during the day at public and private schools located in urban and rural areas of Brazil. The sample was designed to represent this population in 32 geographic strata: each of the 26 capitals and the Federal District (DF), and the five geographic macroregions of the country (North, Northeast, Southeast, South and Central West), constituted by the other counties.^13^

The sample of each stratum was allocated proportionally to the number of schools, according to administrative dependence (private and public). Schools with a total of 15 students or more were eligible according to the 2010 School Census. In the first stage, the schools were selected through probabilities proportional to size (number of students enrolled). In the second stage, ninth grade classes were chosen to be studied in each of the selected schools. All of the 109,104 students from the selected schools, who were present on the day of data collection and who answered the questionnaire, made up the sample. The full description of the sample selection process is available in the PeNSE 2012 scientific paper.^13^

Data collection was performed through a self-administered questionnaire, structured in thematic modules. The questions had multiple choice answers that contained information on sociodemographic, behavioral, eating and health characteristics.^13^

Food consumption was measured by the frequency of food consumption in the seven days prior to the survey date, with responses ranging from daily consumption to no consumption.^13^ Consumption of milk, fresh and minimally processed foods, and ultra-processed foods were evaluated. In addition, we also evaluated the following habits: eating meals while watching TV or studying, having lunch and dinner with guardians, and eating breakfast.^13^ The question regarding milk intake considered the consumption of milk and beverages with milk (coffee or chocolate milk, smoothies and porridge). It did not include yogurt, cheese and other milk derivatives.^13^ Since milk is considered to be the main source of calcium for adolescents, not drinking it on at least one of the seven days was defined as inadequate intake, considering that drinking milk serves more than half of the daily need for calcium. Specifically, a variable was made to represent the consumption of fresh or minimally processed foods (MPF) (beans, raw vegetables, raw salads, cooked vegetables, and fruits). Another variable was made to represent food consumption of ultra-processed foods (UF) (fried salty snacks, sausages, crackers, cookies, snack foods, candies and soda), based on the average number of days consumed in the last week, including the foods defined for each of these categories. Consumption of MPF less than five times a week was attributed to risk behavior, whereas for UF, the risk was considered when they were consumed two or more days in the week. Finally, not having breakfast or meals (lunch and dinner) in the presence of guardians for four days or more of a week was considered a risk behavior.

Physical activity was considered to be a possible factor associated with inadequate milk consumption. For this purpose, the accumulated exercise time in minutes was calculated from the time spent walking and cycling to and from school, physical education at school and general activities such as dance, gymnastics and wrestling. Adolescents with an accumulated activity of less than 300 minutes per week were defined as inactive. Conversely, active adolescents participated in 300 minutes or more of physical activity per week.

Data analysis was performed using Stata 14.0 software. As suggested by the PeNSE methodology, all analyses were performed using the expansion and sample weight technique, according to the selection and population representation process outlined in the research. Descriptive and bivariate analyzes were performed to study the associations. A multiple model was adjusted to independently identify factors associated with inadequate milk consumption.

Data were evaluated for their distribution characteristics. The cutoff points of the eating behavior variables were defined according to frequency of consumption and biological plausibility. The statistical chi-square test was used to measure associations, due to the parametric nature of the variables studied. Subsequently, a logistic regression model was built. In order to select the independent variables eligible to compose the multiple model, the value of p≤0.20 was considered as the inclusion criteria. The variable input technique was *Stepwise Forward,* and the value of p≤0.05 was used to define a statistically significant association.

PeNSE 2012 was approved by the National Research Ethics Commission (*Comissão Nacional de Ética em Pesquisa* - CONEP) of the MS under report no. 16,805. The research participants signed an Informed Consent Form.

## RESULTS

Of the total students analyzed, 82.8% were from public schools and about half were female (52.2%). About a quarter of the adolescents reported being physically active.

Regarding eating behavior, 58.9% of the adolescents showed inadequate milk consumption, with a confidence interval of 95% (95% CI) 55.6-62.1; and 64.4% (95% CI 63.0-65.8) presented UF consumption higher than twice a week. The habit of eating meals while watching TV or studying was present in 81.1% (95% CI 78.7-83.4) of adolescents (Table 1).

**Table 1:** Prevalence and respective confidence intervals of sociodemographic characteristics, physical activity and eating behaviors of Brazilian adolescents. PeNSE 2012.

Sociodemographic characteristics		n	%	95%CI
Macro-region	Southeast	108,828	44.3	31.5-47.1
	Northeast		25.3	23.6-27.0
	North		8.0	7.5-8.5
	South		14.5	13.4-15.8
	Central West		7.9	6.7-9.2
Gender	Female	108,828	52.2	50.2-54.2
	Male		47.8	45.8-49.8
Race	Others^a^	108,765	63.2	57.9-68.2
	White		36.8	31.7-42.1
Age group	≤14 years old	108,828	68.5	59.3-76.4
	>14 years old		31.5	23.6-40.7
Type of school	Public	108,828	82.8	77.7-86.9
	Private		17.2	13.0-22.3
Mother’s education level	Did not study	90,398	10.0	8.4-12.0
	Studied^b^		90.0	88.0 -91.6
Father’s education level	Did not study	83,868	15.2	13.1-17.4
	Studied^b^		84.9	82.6-86.9
Lives with both parents	Yes	108,668	62.2	60.2-64.1
	No		37.8	35.8-39.8
Lives with one of the parents	Yes	108,804	94.6	94.1-95.0
	No		5.4	4.9-5.8
Physical activity				
Accumulated physical activity	Active	108,828	26.6	25.9-27.2
	Inactive		73.4	72.8-74.1
Food behavior				
Milk consumption in the last week	Daily	108,828	41.1	37.9-44.4
	≤6 days		58.9	55.6-62.1
Frequency of eating breakfast	≤4 days	108,730	38.1	35.7-40.5
	>4 days		61.9	59.5-64.3
Consumption of MPF in the last week	<5 days	108,366	82.3	81.2-83.4
	≥5 days		17.7	16.6-18.8
Consumption of UF in the last week	>2 days	108,095	64.4	63.0-65.8
	≤2 days		35.6	34.2-37.0
Eats lunch or dinner with a guardian	≤4 days	108,764	28.5	26.5-30.5
	>4 days		71.5	69.5-73.5
Eats watching TV or studying	Yes	108,706	81.1	78.7-83.4
	No		18.9	16.6-21.3

^a^Dark-skinned black, light-skinned black, Asian, and indigenous; ^b^some level of schooling; OR: odds ratio; 95%CI: 95% confidence interval; MPF: fresh and minimally processed foods; UF: ultra-processed foods.

Table 2 shows the bivariate analysis of factors associated with inadequate milk consumption and their respective prevalence and effect measures. The 15 characteristics tested showed a statistically significant association with this outcome.

**Table 2:** Prevalence, odds ratio and respective confidence intervals of the factors associated with inadequate consumption of milk in Brazilian adolescents. PeNSE 2012.

Characteristics	Inadequate Consumption of Milk (%)	OR (95%CI)	p-value
Macro-region			
Southeast	53.6	1	
Northeast	65.7	1.66 (1.38-2.00)	<0.001
North	58.9	1.24 (1.02-1.51)	0.032
South	52.1	0.94 (0.74-1.20)	0.639
Central West	60.8	1.34 (0.97-1.86)	0.074
Gender			
Female	61.4	1.20 (1.15-1.25)	<0.001
Male	57.0		
Race			
White	55.3	1.29 (1.22-1.37)	<0.001
Others^a^	61.5		
Age			
≤14 years old	57.7	1.23 (1.17-1.28)	<0.001
>14 years old	62.7		
Type of school			
Public	54.5	1.28 (1.20-1.37)	<0.001
Private	60.6		
Mother’s educational level			
Studied^b^	58.3	1.50 (1.40-1.61)	<0.001
Did not study	67.8		
Father’s educational level			
Studied^b^	57.9	1.54 (1.44-1.65)	<0.001
Did not study	67.9		
Lives with both parents			
No	60.7	1.10 (1.07-1.13)	<0.001
Yes	58.4		
Physical activity			
Active	13.8	1.49 (1.45-1.52)	<0.001
Inactive	45.1		
Eats breakfast			
≤4 days	71.2	2.30 (2.17-2.44)	<0.001
>4 days	51.8		
Consumes MPF			
<5 days	62.1	2.10 (1.80-2.47)	<0.001
≥5 days	43.8		
Consumes UF			
>2 days	56.5	0.73 (0.70-0.76)	<0.001
≤2 days	64.0		
East lunch or dinner with guardian			
≤4 days	63.0	1.24 (1.18-1.30)	<0.001
>4 days	57.8		
Eats meals watching TV or studying			
Yes	60.1	1.19 (1.13-1.24)	<0.001
No	56.0		
Asthma			
Yes	57.7	0.93 (0.89-0.97)	0.001
No	59.6		

^a^Dark-skinned black, light-skinned black, Asian, and indigenous; ^b^some level of schooling; OR: odds ratio; 95%CI: 95% confidence interval; MPF: fresh and minimally processed foods; UF: ultra-processed foods.

**Graph 1:**
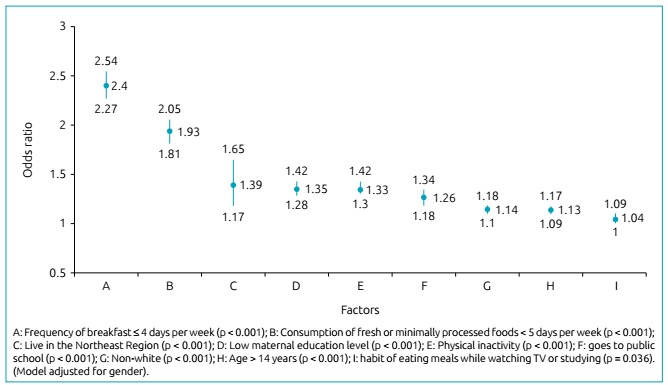
Multiple Logistic model of factors associated with inadequate milk consumption in Brazilian adolescents (n = 89.875) - PeNSE 2012.

After adjusting the logistic model, the following variables remained significant: macroregion, type of school, race, age, maternal education, physical activity, MPF consumption, frequency of breakfast and the habit of eating in front of the TV or studying (Graph 1).

## DISCUSSION

According to the criteria used here, inadequate milk consumption was present in most of the sample studied. Nine factors independently associated with this dietary error were identified: eating breakfast less than four days per week; eating MPF less than five days per week; residing in the Northeast Region; less maternal education; physical inactivity; enrolling in a public school; not being of the white race; being over 14 years old and having the habit of eating meals while watching TV or studying.

Although there are several ways to assess milk consumption, food frequency analysis has been the most widely used in population studies. The literature has identified a significant reduction in the intake of this food in adolescents. This phenomenon has been associated with increased consumption of soft drinks and other ultra-processed beverages, socioeconomic status, gender, age group, eating behavior and nutritional status.^8^ ^,^ ^9^ ^,^ ^10^ ^,^ ^14^ ^,^ ^15^ ^,^ ^16^

Among these factors, we highlight the high consumption of drinks with high sugar content, as they contribute to excessive caloric intake and negatively affect anthropometric parameters, which contribute to excess weight gain.^17^ According to the EYHS, the daily intake of 100g of sugary beverages was associated with increased body mass index (BMI) and adiposity in children aged 9 to 15 years old. In addition, replacing these beverages with milk and water during follow-up was associated with less body fat gain, and reduced BMI and waist circumference.^12^ However, no association was identified in the studied sample between inadequate milk consumption and higher frequency of UF consumption, which included soda. Such disagreement can be explained by the grouping of ultra-processed foods into one composite variable and the way the PeNSE data were obtained, which limits the scope of information related to food consumption to frequency in the last seven days.

Breakfast is a main daily meal.^1^ Not eating it results in lower average macro and micronutrient intake.^18^ Specifically, breakfast, in many cultures, means significant milk consumption,^2^ and a reduction in its frequency may contribute to low milk intake, which may lead to a greater risk of inadequate milk intake among PeNSE adolescents who ate breakfast less frequently.

Although there is an inverse association between age and breakfast,^18^ ^,^ ^19^ the present study identified an association between older age and inadequate milk consumption, regardless of breakfast. This aspect can be explained by the fact that older adolescents consume less milk due to the fact that they consume more soft drinks.^16^

In this regard, adolescents’ food choices are influenced by their social, cultural and media environment, which leads to greater consumption of low-quality nutritional foods, typical for adolescents.^20^ Thus, MPFs are potentially consumed less, meaning that milk is replaced by other beverages. This is in agreement with the association of lower frequency of MPF consumption with lower milk consumption, as evidenced in the studied sample.

Watching TV for extended periods has been linked to unhealthy food consumption.^21^ ^,^ ^22^ Among teenagers, soft drinks and other types of industrialized beverages are among the most consumed foods while watching TV.^11^ ^,^ ^23^ This practice partly supports the growing trend of substituting milk for other processed and sugar-rich drinks.^10^ Thus, excessive exposure to electronic screens represents a higher risk of inadequate milk consumption, as identified in this survey of adolescents who participated in PeNSE 2012.

Physical inactivity was also associated with inadequate milk consumption in adolescents. In fact, physical inactivity has been associated with the consumption of low nutritional quality and high energy density foods.^11^ ^,^ ^24^ Thus, physical inactivity affects food choices, contributes to less dietary variety and, consequently, less essential nutrients, as demonstrated by inadequate intake of milk and dairy products, legumes, fruits, meat, vegetables and cereals.^24^ ^,^ ^25^

As for Brazilian macroregions, adolescents from schools in the Northeast Region had a higher risk of inadequate milk consumption. Despite the possible influence of cultural factors that differ between Brazilian regions, this finding is probably related to the lower purchasing power and lower socioeconomic development of this region,^26^ as shown by the results of the Family Budget Survey (Pesquisa de Orçamentos Familiares - POF) from 2008-2009, which showed that the annual purchase per capita of pasteurized and fresh milk in the Northeast is comparable to about 50% in the Southeast.^6^

In this context, it is worth mentioning the existence of social and economic inequality between races. According to the IBGE, the population that identifies as dark-skinned or light-skinned black has lower income to provide for their vital needs and is exposed to a higher degree of food insecurity.^26^ This statement supports the association of the risk of inadequate milk consumption among adolescents of other races, when compared to the white race. Moreover, although further studies are needed to assess the influence of cultural aspects, this observed difference in consumption between races may be the effect of such conceptions on eating habits, which are generally varied among them.

Additionally, the lower educational level of mothers of adolescents from the PeNSE 2012 was associated with inadequate milk consumption. This finding is similar to that found by a study with data from PeNSE 2009, which showed an association between higher levels of maternal education and regular milk consumption (minimum of five days per week).^14^

Parents/guardians’ education is a determining factor in their children’s behavior and food intake.^9^ ^,^ ^27^ ^,^ ^28^ Data from the Adolescent Cardiovascular Risk Study (*Estudo de Riscos Cardiovasculares em Adolescentes* - ERICA) demonstrated a significant association between low maternal education and unhealthy eating behaviors.^28^ Thus, a mother’s lower level of education leads to greater difficulty in perceiving and assimilating food quality and potentially results in inadequate eating habits.^27^

While the National School Feeding Program (*Programa Nacional de Alimentação Escolar* - PNAE) provides guidelines for healthy eating in public schools based on students’ nutritional needs, which include the provision of at least two days a week for adolescents,^29^ PeNSE students enrolled in these schools had a higher risk of inadequately consuming milk compared to those from private schools. This fact suggests that the goals set by this program may not be fully achieved, especially in municipalities with less socioeconomic resources. Indeed, it has been shown that public school students exhibit less healthy eating behaviors, are more prone to micronutrient deficiency, and consume less milk and dairy products.^9^ ^,^ ^28^ ^,^ ^30^ This circumstance is possibly the result of families of adolescents in public schools’ greater social vulnerability and less access to food.

It is worth noting that the use of secondary data limited the analyses performed in the present study, which used only the information available in the PeNSE 2012 database. Similarly, the fact that data was collected through a self-administered questionnaire potentially led to completion errors and missing information, which could be associated with inadequate milk consumption (nutritional status and consumption of other sugary drinks besides soda).

In addition, frequency-based food consumption assessment made it impossible to estimate the amount of food consumed by adolescents. Specifically, available information on milk made it impossible to estimate total weekly intake in isolation, since milk intake was also estimated when milk was associated with ultra-processed foods, which should not potentially be considered indicators of healthy eating.

On the other hand, PeNSE 2012 is a survey of the population of adolescents enrolled in the ninth grade in Brazil. It used a careful selection process of the participating schools and, consequently, enabled the recruitment of a representative sample of the national territory. Therefore, PeNSE stands out as the only study to evaluate milk consumption among adolescents all around Brazil. In addition, the statistical analysis performed considered the control of confounding factors of associations through a multiple model. It identified the independent effect of the nine factors associated with inadequate milk consumption.

Finally, inadequate milk consumption is prevalent among Brazilian adolescents. The identification of associated factors suggests the need to improve existing government strategies, such as the PNAE and the School Health Program (*Programa Saúde na Escola* - PSE), with the inclusion of actions to encourage guardian education, the practice of physical activity and the promotion of healthy eating habits for teens. Thus, surveillance and guidance actions for the control and prevention of insufficient milk consumption and, consequently, the guarantee of calcium supply, should prioritize sedentary residents, those living in the Northeast Region, non-white people, adolescents enrolled in public institutions, mothers that have a lower level of education, and adolescents that have a habit of not eating breakfast and eat little MPF
